# *Mycobacterium leprae* Activates Toll-Like Receptor-4 Signaling and Expression on Macrophages Depending on Previous Bacillus Calmette-Guerin Vaccination

**DOI:** 10.3389/fcimb.2016.00072

**Published:** 2016-07-08

**Authors:** Anastasia Polycarpou, Martin J. Holland, Ioannis Karageorgiou, Ayad Eddaoudi, Stephen L. Walker, Sam Willcocks, Diana N. J. Lockwood

**Affiliations:** ^1^Department of Clinical Research, Faculty of Infectious and Tropical Diseases, London School of Hygiene & Tropical MedicineLondon, UK; ^2^Molecular and Cellular Immunology Unit, Institute of Child Health, University College LondonLondon, UK

**Keywords:** bacillus Calmette-Guerin, macrophages, *Mycobacterium leprae*, signaling, Toll-like receptor-4, trained immunity, leprosy

## Abstract

Toll-like receptor (TLR)-1 and TLR2 have been shown to be receptors for *Mycobacterium leprae* (*M. leprae*), yet it is unclear whether *M. leprae* can signal through alternative TLRs. Other mycobacterial species possess ligands for TLR4 and genetic association studies in human populations suggest that people with TLR4 polymorphisms may be protected against leprosy. Using human embryonic kidney (HEK)-293 cells co-transfected with TLR4, we demonstrate that *M. leprae* activates TLR4. We used human macrophages to show that *M. leprae* stimulation of cytokine production is diminished if pre-treated with TLR4 neutralizing antibody. TLR4 protein expression was up-regulated on macrophages derived from non-bacillus Calmette-Guerin (BCG) vaccinated healthy volunteers after incubation with *M. leprae*, whereas it was down-regulated in macrophages derived from BCG-vaccinated donors. Finally, pre-treatment of macrophages derived from BCG-naive donors with BCG reversed the effect of *M. leprae* on TLR4 expression. This may be a newly described phenomenon by which BCG vaccination stimulates “non-specific” protection to the human immune system.

## Introduction

*Mycobacterium leprae* is an acid-fast intracellular Gram-positive bacillus, which shows tropism for macrophages and Schwann cells. Leprosy is the chronic granulomatous disease caused by *M. leprae* which mainly affects the skin and peripheral nerves (Britton and Lockwood, 2004). Although *M. leprae* has undergone genome decay and contains large number of pseudogenes (Cole et al., 2001), comparative genomics revealed genetic diversity to be exceptionally rare among four different *M. leprae* strains, showing remarkable conservation of the genome (99.995% identity; Singh and Cole, 2011). This suggests that the pathology of leprosy is determined by the host immune response to *M. leprae*, resulting in a clinical range from tuberculoid leprosy through borderline forms to lepromatous leprosy as classified by Ridley and Jopling (1966). Patients with localized tuberculoid leprosy have few skin lesions, are characterized by normal cell-mediated immune response, antibodies against *M. leprae* are not significantly produced and have very few or no bacilli present in the lesions (Ridley and Jopling, 1966). Patients with lepromatous leprosy have defective cell-mediated immune response (T cell anergy to *M. leprae* antigens; Godal et al., 1971) and produce large amounts of antibodies that are unable to control the multiplication of *M. leprae* in multiple skin lesions (Ridley and Jopling, 1966).

Toll-like Receptors (TLRs) are a family of pattern-recognition receptors that recognize evolutionarily conserved microbial ligands that form part of the first line of defense against infections (Medzhitov et al., 1997). TLRs play a role in the granulomatous inflammation of leprosy (Krutzik et al., 2003). TLR2-TLR1 heterodimers are considered to mediate cell activation by *M. leprae* (Krutzik et al., 2003). The *M. leprae* genome has been scanned to reveal 31 lipoproteins that could serve as pathogen-associated molecular patterns to be recognized by TLR2-TLR1 heterodimers (Krutzik et al., 2003). Synthetic lipoproteins activated monocytes and monocyte-derived dendritic cells through TLR2 (Krutzik et al., 2003). In addition, TLR1 and TLR2 have been shown to be more highly expressed in skin lesions of patients with tuberculoid leprosy than those with lepromatous leprosy (Krutzik et al., 2003). The local expression of the type-1 cytokines such as IFN-γ and IL-12 predominates in tuberculoid lesions, whereas the type-2 cytokines IL-4 and IL-10 characterize lepromatous leprosy lesions (Yamamura et al., 1991). Therefore, the local cytokine environment regulates the expression of TLR2 and TLR1 (Krutzik et al., 2003), possibly determining the outcome of the innate immune response against *M. leprae* infection.

Although in the study by Krutzik *et al.* the TLRs being activated and regulated in leprosy were reported to be TLR2 and TLR1, and the response through TLR4 was described as weak, however, TLR4 and TLR9 have also been implicated in recognizing proteins from other mycobacteria such as *Mycobacterium tuberculosis* (Means et al., 1999, 2001; Jung et al., 2006), *M. paratuberculosis* (Byun et al., 2012) and *M. bovis* bacillus Calmette-Guerin (BCG; Fremond et al., 2003). A genetic association study using a cohort of 441 Ethiopian leprosy patients and 197 healthy controls reported that two single nucleotide polymorphisms of *TLR4* (896G → A and 1196C → T) were associated with protection against leprosy (Bochud et al., 2009). These two polymorphisms have been shown to be associated with lipopolysaccharide (LPS) hyporesponsiveness (Arbour et al., 2000), by causing local conformational changes that may affect ligand binding, folding efficiency, cell surface expression, and protein stability (Ohto et al., 2012). The TLR4 896 G → A mutation may affect the interaction of TLR4 with myeloid differentiation factor (MyD88) and TIR-domain-containing adapter-inducing interferon-β (TRIF) (Figueroa et al., 2012). In addition, these mutations reduce the levels of functional TLR4 expression (Prohinar et al., 2010).

Previously, we have shown that gene and protein expression of TLR2 and TLR4 in skin lesions of Type 1 Reactions, an inflammatory complication that borderline leprosy patients may develop affecting skin lesion and nerves, is significantly reduced during effective corticosteroid treatment (Walker et al., 2012). This indicates a possible role of TLR4 in leprosy and leprosy reactions. Although this has been speculated in the past (Hart and Tapping, 2012), based on previous *M. tuberculosis* research findings, no research study has addressed this hypothesis for *M. leprae* as yet.

Using primary macrophages from TLR4-deficient mice, it has been demonstrated that the induction of TNF by *M. bovis* BCG is mediated partly by functional TLR4 (Fremond et al., 2003). Infection of TLR4 mutant mice with BCG showed that TLR4 signaling may have a critical function in fine tuning of inflammation during chronic mycobacterial infection (Fremond et al., 2003). In addition, BCG vaccination may be responsible for epigenetic modifications of innate immune cells causing a phenomenon described as trained immunity, an imprinting “memory” effect which could persist up to a year after BCG vaccination (Kleinnijenhuis et al., 2012, 2014).

We therefore investigated whether TLR4 may act as a cell receptor for *M. leprae* and whether binding to TLR4 leads to signal transduction and downstream activation of innate immune response pathways. TLR4 is the only TLR that utilizes both MyD88-dependent and independent (TRIF) pathways for signaling (Hoebe et al., 2003; Yamamoto et al., 2003). The classic ligand for TLR4 is LPS of the outer membrane of Gram-negative bacteria (Poltorak et al., 1998). We hypothesized firstly that *M. leprae* activates TLR4, by containing as yet uncharacterized ligands for TLR4 to those already described in other pathogens, and secondly that treatment of macrophages with *M. leprae* could modify the expression of TLR4 depending on previous BCG-vaccination. We used the human embryonic kidney (HEK) 293 Blue TLR4 cell line and monocyte-derived macrophages from BCG-vaccinated and non-BCG vaccinated healthy volunteers to test our hypotheses.

## Materials and methods

### Human samples

Participants were part of a register of healthy volunteers working at the London School of Hygiene & Tropical Medicine. All individuals provided written informed consent. Whole blood was collected by an experienced phlebotomist. Each of the healthy volunteers was classified as BCG-vaccinated or non-BCG-vaccinated after questioning. The vast majority of the BCG-vaccinated healthy volunteers were British and were vaccinated during infancy. Although the BCG status was self-reported, BCG scars were confirmed by the phlebotomist. None of the healthy volunteers had a history of mycobacterial disease. All our volunteers were healthy and were not using any immunosuppressive or other medication. The BCG-vaccinated and non-BCG vaccinated groups were matched for age and sex. The study was approved by the Ethics committee of the London School of Hygiene & Tropical Medicine (Ethics Ref: 7090).

### *M. leprae*, BCG, and TLR ligands

Gamma-irradiated killed *M. leprae* was obtained from Dr. Tom Gillis (National Hansen's Disease Programs, Baton Rouge, Louisiana, USA). The *M. leprae* had been propagated in nude mice, the tissues were NaOH-treated to remove host tissue contamination. All the Multiplicities of Infection (MOIs) of the *M. leprae* used for stimulations were tested for endotoxin contamination with the Pierce LAL chromogenic endotoxin quantitation kit (Rockford, USA) and were all below the limit of detection of this assay. Live BCG Danish strain SSI was donated by Dr Helen Fletcher (London School of Hygiene & Tropical Medicine). Ultrapure LPS from *Escherichia Coli* K12 strain (InvivoGen, San Diego, USA) was used as a positive control ligand for TLR4.

### Cell culture of HEK-Blue-human TLR4 cells

HEK 293 Blue human TLR4 (HEK-Blue-hTLR4) cells were purchased from InvivoGen (San Diego, USA). These HEK-293 cells were engineered to stably express human TLR4, MD2/CD14 co-receptor genes that are linked to an embryonic alkaline phosphatase (SEAP) reporter gene resulting in secretion of SEAP into the tissue culture supernatant when the TLR4, MD2/CD14 receptors are ligated. These cells express low endogenous levels of TLR3 and TLR5. However, TLR3 is a receptor for viral RNA molecules and TLR5 is a receptor for flagellated bacteria, therefore these two receptors were not blocked with any neutralizing antibodies, since neither *M. leprae* nor BCG contain ligands for these receptors. Cells were grown in standard Dulbecco's modified Eagle's medium (DMEM) with 10% fetal bovine serum (FBS) supplemented with 50 U/ml penicillin, 50 μg/ml streptomycin, 100 μg/ml normocin and 2 mM L-glutamine. When cells were 70–80% confluent, they were detached by tapping the flask, counted, and suspended at ~1.4 × 10^5^ cells per ml in medium containing 10% heat-inactivated bovine serum. Cells were seeded at ~2.5 × 10^4^ cells per well, incubated with DMEM medium alone (control) or medium containing increasing concentrations of killed *M. leprae* for 24 h at 37⋅C in a CO_2_ incubator. Twenty microliters of cell supernatant was added to 180 μl of QUANTI-Blue (InvivoGen, San Diego, USA) per well of a flat-bottom 96-well plate and incubated for 1–3 h. SEAP levels were quantified by reading the absorbance at 630 nm using a spectrophotometer (MRXII, DYNATECH).

### Antibodies

Mouse anti-human TLR4 neutralizing monoclonal antibody [clone W7C11, immunoglobulin G1 (IgG1)] was obtained from InvivoGen (San Diego, USA) and used at a final concentration of 1 μg/ml.

### Isolation of human peripheral blood mononuclear cells

Venous blood was drawn from healthy volunteers into sterile blood collection tubes containing heparin, and peripheral blood mononuclear cells (PBMC) were isolated by density centrifugation using Ficoll-Paque (GE Healthcare Life Sciences, USA).

### Culture of human PBMC, differentiation into macrophages and stimulations with killed *M. leprae*

Isolated PBMC were incubated overnight at 37⋅C in RPMI medium supplemented with 0.5 U/ml penicillin, 0.5 μg/ml streptomycin, 10% heat-inactivated fetal bovine serum and human recombinant Macrophage-Colony Stimulating Factor (M-CSF; Multinyi Biotec, Cologne, Germany) at a final concentration of 100 ng/ml and then non-adherent cells were removed by pipetting off the supernatant. Adherent cells were washed with Phosphate Buffered Saline (PBS) and detached by incubation with Accutase enzyme cell detachment medium (eBioscience, San Diego, USA) and scraping when necessary. Cells were then plated in 48-well plates at a concentration of 1 × 10^6^ monocytes/ml in fresh RPMI medium as described above. Cultures were incubated at 37⋅C in a humidified, 95% Air/5% CO_2_ environment for 7 days in order to differentiate into macrophages. After 7 days the complete culture medium was removed and replaced with serum-free RPMI. Cells were rested for 12 h, before pre-treatment for 2 h with mouse anti-human TLR4 neutralizing monoclonal antibody (Invivogen, USA) followed by stimulation with killed *M. leprae* at multiple MOIs or LPS for 24 h. The following day cell supernatants were collected for enzyme-linked immunosorbent assay (ELISA) and stored at −20⋅C. These primary macrophages differentiated from human PBMC were stimulated with multiple MOIs of killed *M. leprae* because the number of MOIs which would lead to an increase of the cytokine production was not known. MOIs > 25 were discontinued because they caused desensitization of the receptor as demonstrated by a reduction in cytokine production. All the different MOIs of the same donor were performed during the same experiment for consistency and the maximum number of healthy volunteers per experiment was three.

### Enzyme-linked immunosorbent assay (ELISA)

Sandwich ELISAs were used for detection of IL-6 (ebioscience, USA), TNF-α (ebioscience, USA), and CXCL10 (R&D systems, USA) in culture supernatants. Assays were performed as recommended by the manufacturers. Cytokine concentrations were determined using standard curves generated from recombinant cytokines. Triplicate wells were used for the assays.

### Fluorescent activated cell sorting

PBMC isolated from non-BCG vaccinated healthy volunteers as described above were treated with red blood cell (RBC) lysis buffer (Becton Dickinson, USA). Cells were washed, filtered and counted and extracellular staining was performed with mouse anti-human monoclonal CD3-PerCPCy5.5 (eBioscience, San Diego, USA) and mouse anti-human CD14-Alexa Fluor 488 (Becton Dickinson, USA) in PBS supplemented with 2% FBS and EDTA 0.8 mM for 25 min at +4⋅C. The cells were processed through either BC Moflo XDP (Beckman Coulter, California) or BD FACSAriaIII (Becton Dickinson, San Jose, California) high speed cell sorters at Flow Cytometry Core Facility at the Institute of Child Health, University College London. Sorting the monocyte population from one sample required at least 3 h using any of the two cell sorters and therefore in order to process samples from two healthy volunteers during every experiment, we used concurrently both cell sorters. Monocytes were considered the cells which were CD3^−^CD14^+^. Single colored-stained CD3^+^ and CD14^+^ cells were used as controls for gating the CD3^−^CD14^+^ monocytes. Single stained CD3^+^ and CD14^+^ CompBeads (Becton Dickinson, USA) were used as compensation controls.

### Culture of monocytes sorted by flow cytometry, differentiation into macrophages and stimulation with BCG and/or *M. leprae*

Flow cytometry cell-sorted monocytes were plated in T75 flasks containing RPMI medium supplemented with 20% FBS, 0.5 U/ml penicillin, 0.5 μg/ml streptomycin, and human recombinant M-CSF (Multinyi Biotec, Cologne, Germany) at a final concentration of 100 ng/ml and incubated at 37⋅C for 7 days until differentiation. Non-adherent cells were removed by pipetting the supernatant. Adherent macrophages were detached using Accutase enzyme cell detachment medium (eBioscience, San Diego, USA) and washed. Macrophages were then re-plated in 24-well plates at a concentration of 2.5 × 10^5^ macrophages/ml in fresh RPMI supplemented with 5% heat-inactivated FBS and left to rest for 2 h. Half of the macrophages from each donor remained unstimulated and the other half were stimulated with *BCG* at MOI = 10 for 18 h. After 18 h the culture media was replaced with fresh RPMI supplemented with 5% FBS and left to rest for 8 h. Both the untreated and the *BCG*-treated macrophages were subsequently treated or not with killed irradiated *M. leprae* at MOI = 5 or MOI = 10 or LPS 100 ng/ml for 18 h. Adherent macrophages were removed by addition of Accutase enzyme cell detachment medium (eBioscience, San Diego, USA) for 25 min followed by washing and they were transferred into 5 ml Falcon tubes for multicolor flow cytometry staining.

### Multicolor flow cytometry

Detached macrophages in 5 ml Falcon tubes were centrifuged at 800 g for 10 min. Supernatant was decanted and the cell pellet re-suspended in PBS/10% human AB serum for 10 min. Extracellular staining was performed using mouse anti-human CD3-PerCPCy5.5 (Becton Dickinson; USA), mouse anti-human CD14-V450 (Becton Dickinson; USA), mouse anti-human CD16-APC-Cy7 (Becton Dickinson; USA), and live/dead stain (FITC; Invitrogen; USA) in FACS buffer (filter sterilized PBS containing 2% NaN_3_ and 1% heat-inactivated FBS) for 25 min at +4⋅C. Macrophages were fixed with 4% Paraformaldehyde for 15 min at +4⋅C. Cells were permeabilized using PermWash solution (Becton Dickinson; USA) for 15 min at Room Temperature (RT) followed by intracellular staining with mouse anti-human CD68-APC (Biolegend; San Diego, USA) and mouse anti-human TLR4-PE (ebioscience; San Diego, USA) for 25 min at +4⋅C. Cells were suspended in FACS buffer before acquiring at least 100,000 events (singlet cells) on a LSRII flow cytometer (Becton Dickinson; USA). Our panel was multicolor, therefore CompBeads (Becton Dickinson; USA) and Fluorescence minus one (FMO) controls were employed throughout to control for multicolor flow cytometry and for antibody isotype controls (e.g., mouse anti-human TLR4-PE isotype control), which were run on the flow cytometer each time we performed an experiment on the same day as the samples. The FACS gating strategy was: R1 = singlets, cells, live cells, CD3^−^ cells, CD68^+^ cells (macrophages) and TLR4 expression on CD68^+^ macrophages. Two subgroup analyses for TLR4 expression on CD14^+^CD68^+^ macrophages and CD16^+^CD68^+^ macrophages were also performed. Data were analyzed using FlowJo v9 (Tree Star, Oregon, USA). TLR4 expression on CD68^+^ macrophages were displayed as histograms from which the Median Fluorescence Intensity (MFI) was derived. Graphs of MFI and their percentage change compared to unstimulated control (medium) were plotted using GraphPad Prism 6.0.

### Statistical methods

All statistical analyses were performed using GraphPad Prism 6.0. Non-parametric Wilcoxon signed-rank tests and Mann-Whitney tests were used for comparisons. A parametric *t*-test was used for the comparisons of the HEK-Blue-hTLR4 cell line experiment. Values of *p* < 0.05 were considered significant.

## Results

### *M. leprae* binds to TLR4 leading to signal transduction

**Figure 1:**
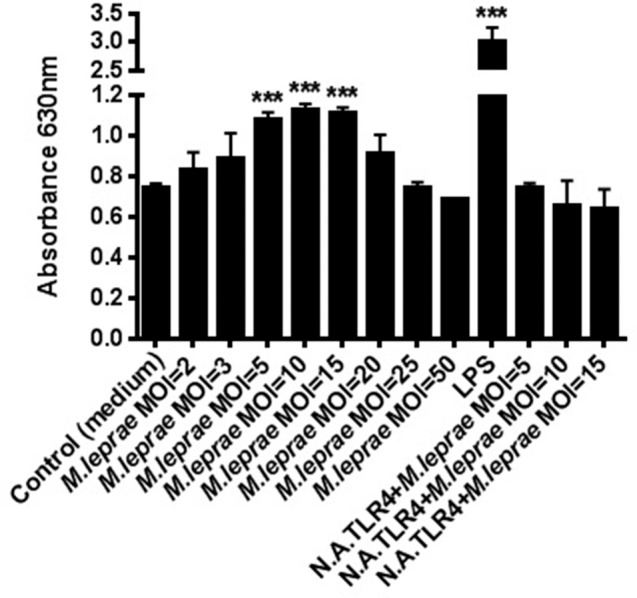
***M. leprae* signals through TLR4**. Human embryonic kidney (HEK) 293-Blue cell line expressing human TLR4, MD2/CD14 co-receptor genes and a secreted embryonic alkaline phosphatase (SEAP) reporter gene, was stimulated with increasing concentrations of killed whole *M. leprae* for 24 h with or without a monoclonal TLR4 neutralizing antibody (N.A.TLR4). The levels of alkaline phosphatase in the supernatants were detected in medium by reading the OD at 630 nm (MOI = Multiplicity of infection). ^***^p ≤ 0.0001 (student's *t*-test) compared to unstimulated control.

To determine whether TLR4 mediates cell activation by *M. leprae*, we used HEK-Blue-hTLR4 cells, co-expressing MD2/CD14, that report signaling through the TLR4 receptor by the secretion of alkaline phosphatase. The cell line was stimulated with irradiated whole *M. leprae* at increasing MOI for 24 h and the levels of alkaline phosphatase in the supernatants were determined. Stimulation of the cell line with *M. leprae* at MOI = 5, MOI = 10, MOI = 15 led to highly statistically significant (*p* ≤ 0.0001) dose-dependent increases in the level of alkaline phosphatase in the supernatants compared to the unstimulated control (medium; Figure 1). This effect was lost when *M. leprae* at high MOI ≥ 20 was tested (Figure 1). HEK293 cells are widely used in innate immunity research as they naturally express very low levels or no TLRs other than the transfected TLR of interest. However, to confirm the specificity of *M. leprae*-TLR4 interaction, we also blocked this receptor with a monoclonal neutralizing antibody. Significant production of alkaline phosphatase in the supernatants seen after stimulation of the cell line with *M. leprae* MOI between 5 and 15 was abolished if the cell line was pre-treated with the monoclonal TLR4 neutralizing antibody, indicating that this was a TLR4 specific effect (Figure 1).

### Pre-treatment with neutralizing antibody for TLR4 reduces the cytokine production by human macrophages

**Figure 2:**
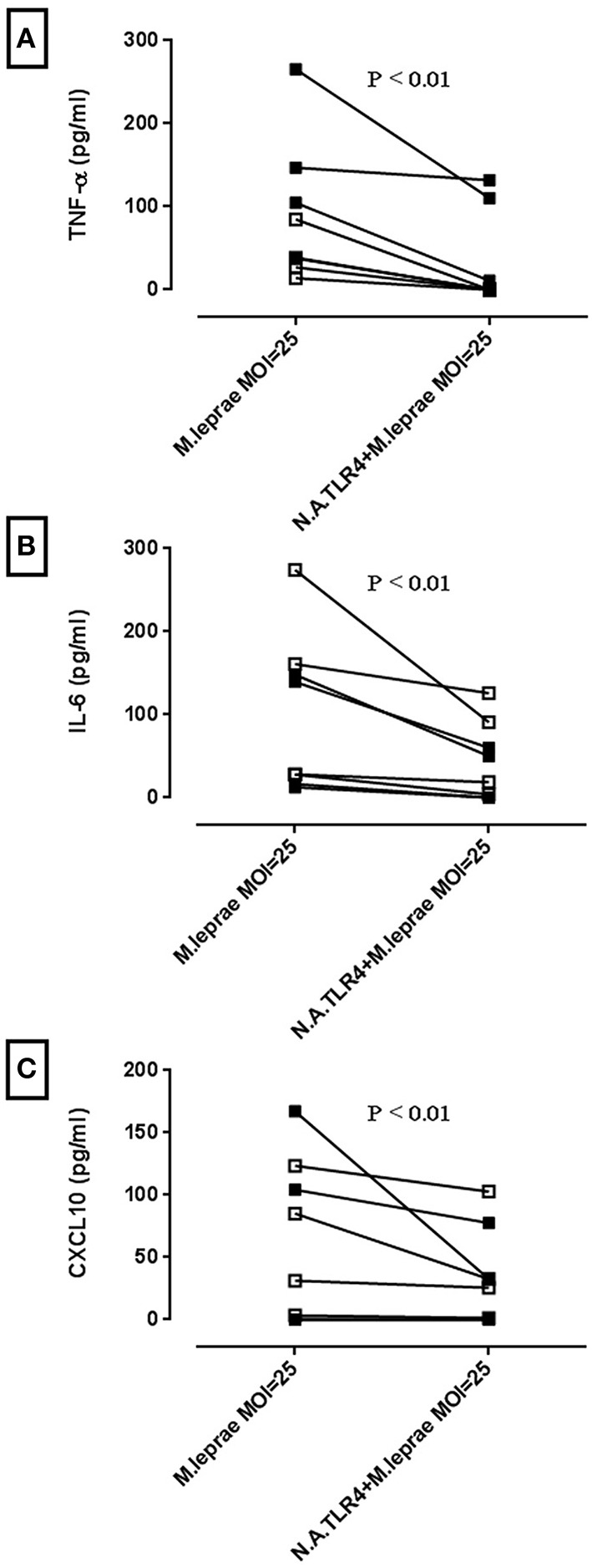
***M. leprae* stimulates the production of pro-inflammatory cytokines in human macrophages partly through TLR4 activation**. Macrophages differentiated from isolated PBMC were treated with neutralizing antibody for TLR4 (N.A.TLR4) for 2 h followed by incubation for 24 h with killed *M. leprae*. Supernatants were collected and ELISAs for pro-inflammatory cytokines TNF-α, IL-6, and CXCL10 were performed. Graphs **(A–C)** are derived from *n* = 4 non-*BCG*-vaccinated healthy volunteers (white squares) and *n* = 4 *BCG*-vaccinated healthy volunteers (black squares). Wilcoxon signed-rank tests were performed for each cytokine and showed significant reductions in cytokine production by anti-TLR4 neutralizing antibody. Median reduction in pg/ml for TNF-α = 38.34 (*P* < 0.01), IL-6 = 29.49 (*P* < 0.01) and CXCL10 = 13.19 (*P* < 0.01).

To test whether activation of TLR4 signaling results in an inflammatory response, human primary macrophages derived from differentiated PBMC of healthy volunteers were incubated with increasing MOI of irradiated whole *M. leprae* for 24 h. The supernatants were collected and ELISAs for both MyD88-dependent cytokines (IL-6 and TNF-α) and the MyD88-independent cytokine CXCL10 (Zughaier et al., 2005), were performed. In macrophages derived from both *BCG*-vaccinated and non-*BCG*-vaccinated healthy volunteers, incubation with *M. leprae* led to a secretion of all three cytokines (Figure 2). Pre-incubation of the macrophages with neutralizing monoclonal antibody for TLR4 before stimulation with *M. leprae*, led to a significant reduction in the three secreted cytokines, indicating that the observed TLR4 signal transduction leads to cytokine production in primary cells (Figure 2).

### Stimulation of human macrophages with *M. leprae* leads to modulation of TLR4 protein expression

**Figure 3:**
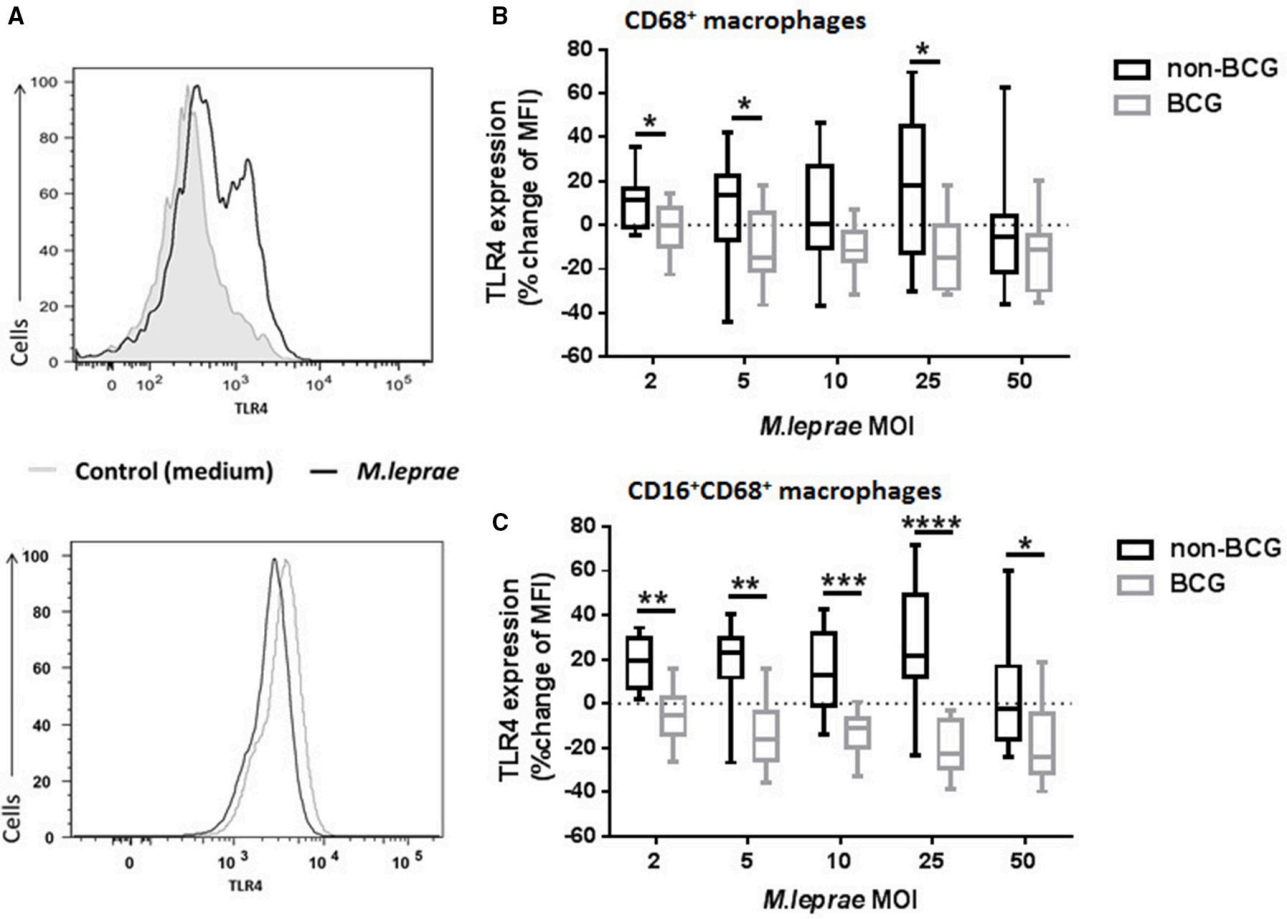
**TLR4 expression in macrophages is modulated in *BCG*-vaccinated and non-*BCG* vaccinated healthy volunteers after exposure to killed *M. leprae***. **(A)** Histograms showing up-regulation of TLR4 expression in macrophages from a non-BCG vaccinated volunteer (upper histogram) and down-regulation of TLR4 expression in macrophages from a BCG-vaccinated healthy volunteer (lower histogram) after incubation with killed *M. leprae* for 18 h. **(B)** Percentage change of Median Fluorescence Intensity (MFI) indicating expression of TLR4 compared to unstimulated control (medium) after incubation of macrophages with increasing concentrations of killed *M. leprae* for 18 h. Using multicolor flow cytometry, macrophages were gated as CD68^+^ cell population. The plot shows the total % change in TLR4 expression compared to controls in macrophages from non-*BCG* vaccinated (black boxes) and *BCG*-vaccinated (gray boxes) healthy volunteers, after incubation with increasing MOI of killed *M. leprae*. **(C)** Subgroup analysis of percentage change of TLR4 MFI for the CD16^+^CD68^+^ macrophage population. Graphs summarize (*n* = 8) experiments with a total of 14 non-*BCG* vaccinated healthy volunteers and 10 *BCG*-vaccinated healthy volunteers. Mann-Whitney tests were used to compare the non-BCG vaccinated with the BCG-vaccinated donors. ^*^0.01 < *p* < 0.05; ^**^0.001 < *p* < 0.01; ^***^0.0001 < *p* < 0.001, ^****^p < 0.0001.

Since expression of TLR by cells has been reported to be affected by engagement with its ligand, we investigated whether TLR4 protein expression would change after overnight incubation with *M. leprae*. BCG contains a TLR4 ligand (Fremond et al., 2003) and therefore macrophages prepared from PBMC of BCG-vaccinated and unvaccinated healthy volunteers were incubated overnight with increasing MOI of killed *M. leprae.* TLR4 expression in CD68^+^ live gated cells was then assessed by FACS analysis (Figure 3A). Permeabilization of the macrophages followed by intracellular staining for TLR4, meant that both intracellular and cell surface levels of TLR4 were detected. The median baseline TLR4 expression in macrophages derived from *BCG*-vaccinated healthy volunteers was similar to the baseline levels of TLR4 expression by macrophages derived from non-*BCG* healthy volunteers (data not shown). We observed an increase in TLR4 MFI (maximum increase of 69.71% with *M. leprae* of MOI = 25) in macrophages from non-*BCG*-healthy volunteers (*n* = 14) after overnight incubation with *M. leprae* (this effect was not seen at MOI = 50; Figure 3B). However, we observed a down-regulation of TLR4 (maximum 36.45% after overnight incubation with *M. leprae* MOI = 5) in macrophages from *BCG*-vaccinated healthy volunteers (Figure 3B; Supplementary Image 1). The difference in TLR4 expression between *BCG*-vaccinated and non-*BCG*-vaccinated healthy volunteers was also seen when cells were gated on the CD16^+^CD68^+^ macrophages (Figure 3C; Supplementary Image 1).

### Pre-treatment of macrophages with BCG reverses the effect of *M. leprae* on the expression of TLR4

**Figure 4:**
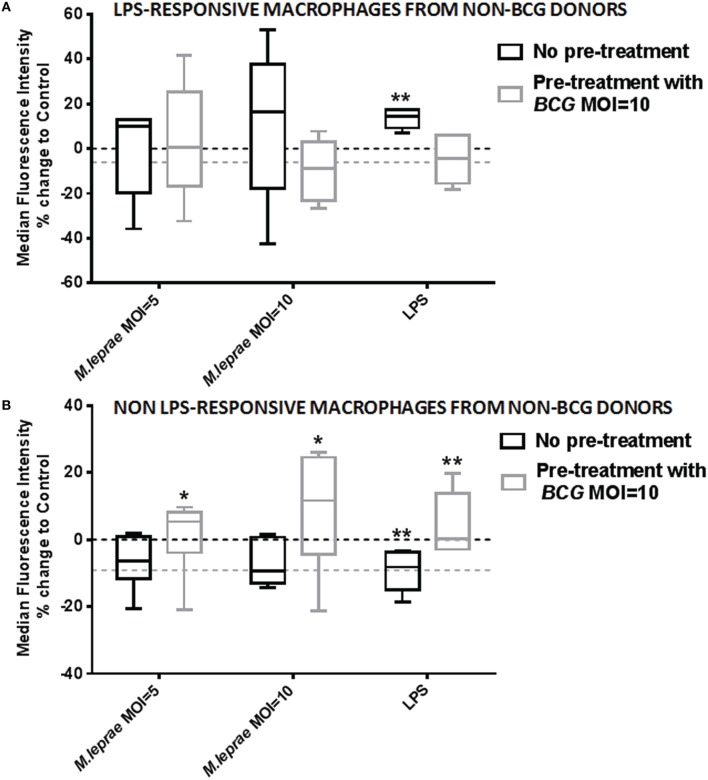
**Exposure to live *BCG* reverses the modulation of TLR4 expression by *M. leprae* in macrophages from non-*BCG* vaccinated healthy volunteers**. Percentage change of Median Fluorescence Intensity (MFI) indicating expression of TLR4 compared to unstimulated control (medium) after incubation of differentiated human macrophages derived from FACS-sorted monocytes with increasing concentrations of killed *M. leprae* or LPS for 18 h. Using multicolor flow cytometry, macrophages were gated as CD68^+^ cell population. The plot shows the total % change in TLR4 expression compared to control (dashed line) in macrophages without pre-treatment with *BCG* (black boxes) and with pre-treatment with *BCG* MOI = 10 for 18 h (gray boxes), following incubation with MOI = 5 and MOI = 10 of killed *M. leprae* or with LPS 100 ng/ml for 18 h. Macrophages showing up-regulation of TLR4 expression after incubation with LPS for 18 h were considered LPS-responsive **(A)**, whereas macrophages showing down-regulation of TLR4 after incubation with LPS were considered non-LPS responsive **(B)**. Graphs summarize a total of five non-*BCG* vaccinated healthy volunteers responsive to LPS **(A)** and a total of six non-*BCG* vaccinated healthy volunteers non-responsive to LPS **(B)**. Wilcoxon signed-rank tests were used to compare the different treatments with unstimulated control (dashed line). ^*^0.01 < *p* < 0.05; ^**^0.001 < *p* < 0.01.

In order to investigate the different effect of killed *M. leprae* in macrophages of BCG-vaccinated and unvaccinated healthy volunteers, we added live BCG to macrophages before incubation with *M. leprae*. Monocytes (CD3^−^CD14^+^ PBMC) from non-BCG healthy volunteers were FACS-sorted to acquire pure macrophage populations after a week of differentiation, without the presence of any T-lymphocytes. The macrophages were either treated with live BCG SSI strain overnight or nothing. The cells were washed to remove BCG non-incorporated by the macrophages and left to rest for 8 h before the addition of killed *M. leprae* or LPS overnight followed by FACS analysis for TLR4 expression in CD68^+^ macrophages. Treatment of macrophages with live *BCG* alone resulted in a clear down-regulation of TLR4 expression compared to unstimulated control (dotted line in Figures 4A,B). However, treatment of macrophages with killed *M. leprae* or LPS led to a heterogeneous result: macrophages from certain donors showed an up-regulation of TLR4 expression, while macrophages of other donors showed a down-regulation of TLR4 expression. Since our healthy volunteers varied in exposure to both genetic and environmental factors which could contribute toward this result, we decided to split them into two groups based on their responsiveness to LPS, which served as our positive control being the classical ligand for TLR4. Therefore, five of the healthy volunteers were considered as LPS-responsive after showing up-regulation of TLR4 expression when incubated overnight with LPS, while six volunteers were considered non-LPS responsive since they showed a down-regulation of TLR4 expression after treatment with LPS. This subgroup analysis demonstrated that treatment of macrophages with killed *M. leprae* shows a similar response to treatment with LPS. Macrophages from LPS-responsive donors showed up-regulation of TLR4 after overnight incubation with *M. leprae* whereas macrophages derived from non-LPS responsive donors showed down-regulation of TLR4 expression. Interestingly, we observed a reversal of this effect when we incorporated the pre-treatment with live BCG into this analysis. The LPS-responsive macrophages after pre-treatment with BCG showed a small down-regulation of TLR4 expression, but when *M. leprae* was also added, they lost their ability to up-regulate TLR4, becoming “unresponsive” in contrast to the macrophages which did not have any BCG pre-treatment (Figure 4A). Consequently, the non-LPS responsive macrophages after treatment with BCG showed a small down-regulation of TLR4 expression, but when killed *M. leprae* was also added, they showed a significant up-regulation of TLR4 becoming “responsive,” in contrast to the macrophages without BCG pre-treatment (Figure 4B). The reversal of the effect of killed *M. leprae* on the TLR4 expression in macrophages by BCG was even more evident when a higher MOI of killed *M. leprae* was used, illustrating a dose-responsive effect (Figures 4A,B).

## Discussion

It has been previously demonstrated that TLR1 and TLR2 heterodimers are activated by *M. leprae* (Krutzik et al., 2003). We undertook this study in order to investigate the possible involvement of TLR4 in infection by *M. leprae*. We have shown that *M. leprae* contains a ligand that activates TLR4 leading to signal transduction. The very low or absent level of TLR2 expression by HEK293 cells suggests that the TLR4/MD2/CD14 complex is necessary and sufficient for this signaling. In addition, we demonstrated that TLR4 is also engaged in *ex-vivo* derived human macrophages, the preferred niche of live *M. leprae*. When using a monoclonal neutralizing antibody against TLR4, the production of pro-inflammatory cytokines TNF-α, IL-6, and CXCL10 after overnight incubation with killed *M. leprae* was consistently diminished. We observed modulation of TLR4 protein expression after stimulating human macrophages with killed *M. leprae*, which appears to be regulated in the opposite direction in *BCG*-vaccinated and non-*BCG* vaccinated healthy volunteers. Finally, when we added live BCG to macrophages derived from non-BCG vaccinated healthy donors followed by addition of killed *M. leprae*, we observed a reversal of the effect of *M. leprae* on the expression of TLR4.

Our finding that *M. leprae* activates TLR4 does not contradict the study by Krutzik et al. which concluded that among the TLR homodimers tested, only TLR2 was able to mediate responsiveness toward *M. leprae* (Krutzik et al., 2003). In fact, their results also indicated a 10-fold increase in TLR4 activity by *M. leprae* in the transfected HEK cells expressing TLR4 homodimers, but this was not described in the results (Krutzik et al., 2003). In addition, our findings may explain the results of a study which showed that *in vitro* stimulation of human monocytes with both live and sonicated *M. leprae* induces interferon (IFN)-β mRNA and protein (Teles et al., 2013). The induction of the MyD88-independent/TRIF-dependent gene IFN-β (Covert et al., 2005; Andersen et al., 2008) further supports the signaling via the TRIF pathway of TLR4 after exposure to *M. leprae*. Teles *et al.* have shown that IFN-β and its downstream genes were preferentially expressed in lepromatous leprosy lesions, whereas IFN-γ was preferentially expressed in tuberculoid lesions (Teles et al., 2013). Although IFN-β was found in macrophages and co-localized with CD14, the presence of TLR4 was not examined (Teles et al., 2013). These authors suggested that the increase of type I interferon might be due to intercurrent viral infections (Teles et al., 2013). A more likely explanation is that signaling through the TLR4/CD14/MD2 complex leads to the increase in local type 1 interferon production. Our results, showing the activation of TLR4 by *M. leprae*, could explain why TLR4, using both MyD88 and TRIF-dependent pathways, induces the secretion of type 1 interferons such as IFN-β. Signaling through TLR4 could shift the immunological balance from localized to disseminated disease in leprosy. If this is the case, then our study may have implications in the treatment of leprosy and other mycobacterial diseases since the induction of IFN-β responses in lepromatous leprosy may be blocked by a treatment strategy that directly inhibits TLR4-TRIF signaling. This may be a more effective strategy favoring appropriate responses that protect against mycobacterial infections.

Although we have demonstrated *M. leprae* signal transduction and activation through TLR4, the *M. leprae* ligand(s) remain to be determined. The classical ligand for TLR4 is the LPS which is abundant on the bacterial cell wall of Gram-negative bacteria (Munford, 2008) and is not found on mycobacteria. However, mycobacterial glycolipids such as the lipooligosaccharides (LOS) contain the lipid A portion, which is responsible for the LPS interaction with TLR4 (Park et al., 2009). LOS derived from non-related bacterial species such as *Campylobacter jejuni* (Stephenson et al., 2013) and *Neisseria meningitidis* (Liu et al., 2010) have been demonstrated to be potent activators of TLR4-mediated immunity. Interestingly, certain mycobacterial species such as *M. marinum* (Burguiere et al., 2005; Rombouts et al., 2009), *M. kansasii* (Hunter et al., 1983), *M. gastri* (Gilleron et al., 1993), and the Canetti variant of *M. tuberculosis* (Daffe et al., 1991) contain LOS.

Other candidate *M. leprae* ligands binding to TLR4 are the mycobacterial proline-glutamate (PE)/proline-proline-glutamate (PPE) proteins. The *pe/ppe* genes are unique to mycobacteria and particularly abundant in pathogenic mycobacteria such as *M. tuberculosis* (Sampson, 2011), whereas PE/PPE proteins of *M. leprae* have been described to be recognized by PBMC from leprosy patients, stimulating IFN-γ production (Choi et al., 2013). The *M. tuberculosis* protein pair PE9 (Rv1088)-PE10 (Rv1089) has recently been reported to physically interact to form heterodimers, and the PE9-PE10 complex binds to and activates TLR4 in human macrophages, resulting in an increase in the transcript levels of IFN-β and inducing macrophage apoptosis (Tiwari et al., 2015). The PE9-PE10 complex is, therefore, a newly described apoptosis-inducing factor which acts through TLR4 and is likely to play a role in the immune evasion strategies of *M. tuberculosis* (Tiwari et al., 2015).

Other molecules on *M. leprae* activating TLR4 could be heat shock protein-65 (HSP-65; Bulut et al., 2005), chaperonin 60 protein (Cehovin et al., 2010), or adhesion heparin-binding hemagglutinin (HBHA; Jung et al., 2011). These three molecules have been shown to mediate TLR4 recognition for *M. tuberculosis* (Bulut et al., 2005; Cehovin et al., 2010; Jung et al., 2011; Basu et al., 2012) and although it has been speculated that *M. leprae* could also possess similar TLR4 stimulating qualities by encoding orthologues of HSP60, HSP65 and HBHA (Hart and Tapping, 2012), the exact ligand on *M. leprae* leading to activation of TLR4 remains elusive.

We observed modulation of TLR4 protein expression in macrophages after incubation with killed *M. leprae* which was differentially expressed in macrophages derived from BCG-vaccinated and non-BCG-vaccinated healthy volunteers. This difference was more obvious when the subgroup analysis of CD16^+^CD68^+^ macrophages was performed. CD16 is a regulator of the TRIF-dependent TLR4 response in human monocytes by activating Syk, IFN regulatory factor 3 and STAT1, resulting in enhanced expression of MyD88-independent genes (Shalova et al., 2012). The general trend of up-regulation of TLR4 expression in macrophages derived from the unvaccinated donors after incubation with low MOI of *M. leprae* provides further support of an important role of TLR4 in leprosy. Interestingly, up-regulation of TLR4 mRNA in mononuclear leukocytes has been described in patients with active pulmonary tuberculosis (Chang et al., 2006). Up-regulation of expression of TLR4 and its co-receptor MD-2 in human mononuclear phagocytes increases responsiveness to LPS through enhanced phosphorylation of downstream signaling molecule IRAK and increase of NF-κB DNA binding activity, leading to enhanced cytokine production (Bosisio et al., 2002). Although the down-regulation of TLR4 expression observed after stimulation with very high MOI of *M. leprae* could be explained as a desensitization of TLR4, it still remains unclear why exposure to low MOI of *M. leprae* in BCG-vaccinated derived macrophages leads to down-regulation of TLR4. Down-regulation of TLR4 has been an attributed mechanism of endotoxin tolerance (Nomura et al., 2000) i.e., macrophages exposed to LPS become hyporesponsive to a second challenge with LPS. Therefore, one explanation is that these donors have been pre-exposed to other TLR4 ligands resulting in LPS-like TLR4 tolerance. Indeed, the down-regulation of TLR4 could be an indication of such an endotoxin tolerance effect due to the early exposure of these donors to the BCG which contains a ligand for TLR4 (Fremond et al., 2003; Nicolle et al., 2004).

Our results may suggest an enduring modification of the donors' innate immune response due to BCG-vaccination early in life, creating an imprinting “memory” effect which persists into adulthood. An explanation of our observations could be via a monocyte-T lymphocyte interaction (heterologous immunity) or alternatively due to “trained” immunity: an immune response describing innate immune responses with enhancing features seen in human monocytes (Kleinnijenhuis et al., 2012) and macrophages (Saeed et al., 2014). BCG may induce epigenetic modifications in monocytes, which can last up to a year after the BCG-vaccination (Kleinnijenhuis et al., 2012, 2014). The chromatin mark H3K4me3 results in higher IL-6, TNF-α and TLR4 expression in monocytes from BCG-vaccinated individuals, compared to monocytes from non-BCG vaccinated individuals for at least 3 months after vaccination (Kleinnijenhuis et al., 2012). In addition, the effects of BCG vaccination on TLR4 expression and LPS-induced pro-inflammatory cytokines are long-standing and present for at least one year (Kleinnijenhuis et al., 2014). BCG vaccination is expected to affect acquired immunity but our data could suggest that some years after vaccination, non-specific effects of BCG vaccination can still be detected. Our result is in agreement with a recent clinical study from Bangladesh in which healthy household contacts of leprosy patients who received BCG vaccination as immunoprophylaxis. Twenty-one (0.4%) of those vaccinated developed clinical manifestations of leprosy within 12 weeks, suggesting that BCG may unmask subclinical leprosy (Richardus et al., 2015). This described phenomenon could arise either from boosting of cell–mediated immunity by homologs of *M. leprae* present in BCG or from epigenetic reprogramming of innate cells described as trained immunity (Kleinnijenhuis et al., 2012).

The monocyte population can turn over quickly (Yona et al., 2013), but this does not explain how “trained” monocytes may be maintained in the human body years after the BCG vaccination. However, cell reprogramming could take place at the level of progenitor cells in the bone marrow and innate immune memory can be transferred via progenitor cells (Yanez et al., 2013). In addition, histone modifications can persist through cell division (Gaydos et al., 2014). Moreover, monocytes differentiating into macrophages undergo substantial epigenetic changes (Saeed et al., 2014) and the lifespan of the macrophage could be months to years (Murphy et al., 2008; Wilson et al., 2010). Interestingly, recent evidence demonstrate that major tissue-resident macrophage populations are established prior to birth and maintain themselves subsequently during adulthood independent of replenishment by blood monocytes (Yona et al., 2013).

Finally, our observation that *ex vivo* treatment of a pure population of non-BCG macrophages with live BCG reverses whatever effect killed *M. leprae* has on the TLR4 expression of these cells, could be another indication that *M. leprae* acts through the same receptor as BCG and LPS. We acknowledge that a limitation of our study is that we did not perform genetic analysis for TLR4 polymorphisms in our group of healthy volunteers. In addition, we were not able to address the effect of latent tuberculosis on our donors, a factor that could also account for macrophages possibly responding in a different way after stimulation with *M. leprae*. Nevertheless, it remains important that the non-LPS responsive macrophages derived from half the donors, which did not respond with an up-regulation of TLR4 expression after contact with *M. leprae*, when pre-exposed to live BCG, were transformed into “responsive” macrophages by up-regulation of their TLR4 expression after exposure to *M. leprae*. The question whether this modulation of TLR4 expression could be another mechanism by which live BCG acts on innate immune cells rendering them more responsive and therefore more equipped to fight future exposure to pathogenic bacteria also acting through TLR4, remains to be further investigated.

## Author contributions

AP, MH, SLW, and SW formulated the hypotheses and designed the study protocol; AP, IK, and AE conducted the laboratory work; AP, MH, and IK analyzed the data; AP drafted the manuscript and is the guarantor of the paper; AP, MH, SLW, and DL contributed to the design and conduct of the laboratory work and critical review of the manuscript. All authors contributed to the interpretation of the data and writing of the manuscript and read and approved the final version.

## Funding

This work was supported by the Hospital and Homes of St Giles (grant numbers ITCRBH15 and ITCRZG69).

### Conflict of interest statement

The authors declare that the research was conducted in the absence of any commercial or financial relationships that could be construed as a potential conflict of interest.

## Supplementary material

Supplementary Image 1 **TLR4 expression in macrophages is modulated in *BCG*-vaccinated and non-*BCG* vaccinated healthy volunteers after exposure to killed *M. leprae***. Percentage change of Median Fluorescence Intensity (MFI) indicating expression of TLR4 compared to unstimulated control (medium) after incubation of macrophages with increasing concentrations of killed *M. leprae* for 18 h. Using multicolor flow cytometry, macrophages were gated as CD68^+^ cell population. The upper scatter plot shows the total % change in TLR4 expression compared to controls in macrophages from non-*BCG* vaccinated (white squares) and *BCG*-vaccinated (black squares) healthy volunteers, after incubation with increasing MOI of killed *M. leprae*. The lower scatter plot shows the subgroup analysis of percentage change of TLR4 MFI for the CD16^+^CD68^+^ macrophage population. Graphs summarize (*n* = 8) experiments with a total of 14 non-*BCG* vaccinated healthy volunteers and 10 *BCG*-vaccinated healthy volunteers. Mann-Whitney tests were used to compare the non-*BCG* vaccinated with the *BCG*-vaccinated donors. ^*^0.01 < *p* < 0.05; ^**^0.001 < *p* < 0.01; ^***^0.0001 < *p* < 0.001; ^****^*p* < 0.0001.
