# Supplementary figures and images for: Mycobacterium leprae Activates Toll-Like Receptor-4 Signaling and Expression on Macrophages Depending on Previous Bacillus Calmette-Guerin Vaccination
Source: Front Cell Infect Microbiol. 2016 Jul 8;6:72. doi: 10.3389/fcimb.2016.00072 (PMC4937034; doi:10.3389/fcimb.2016.00072)

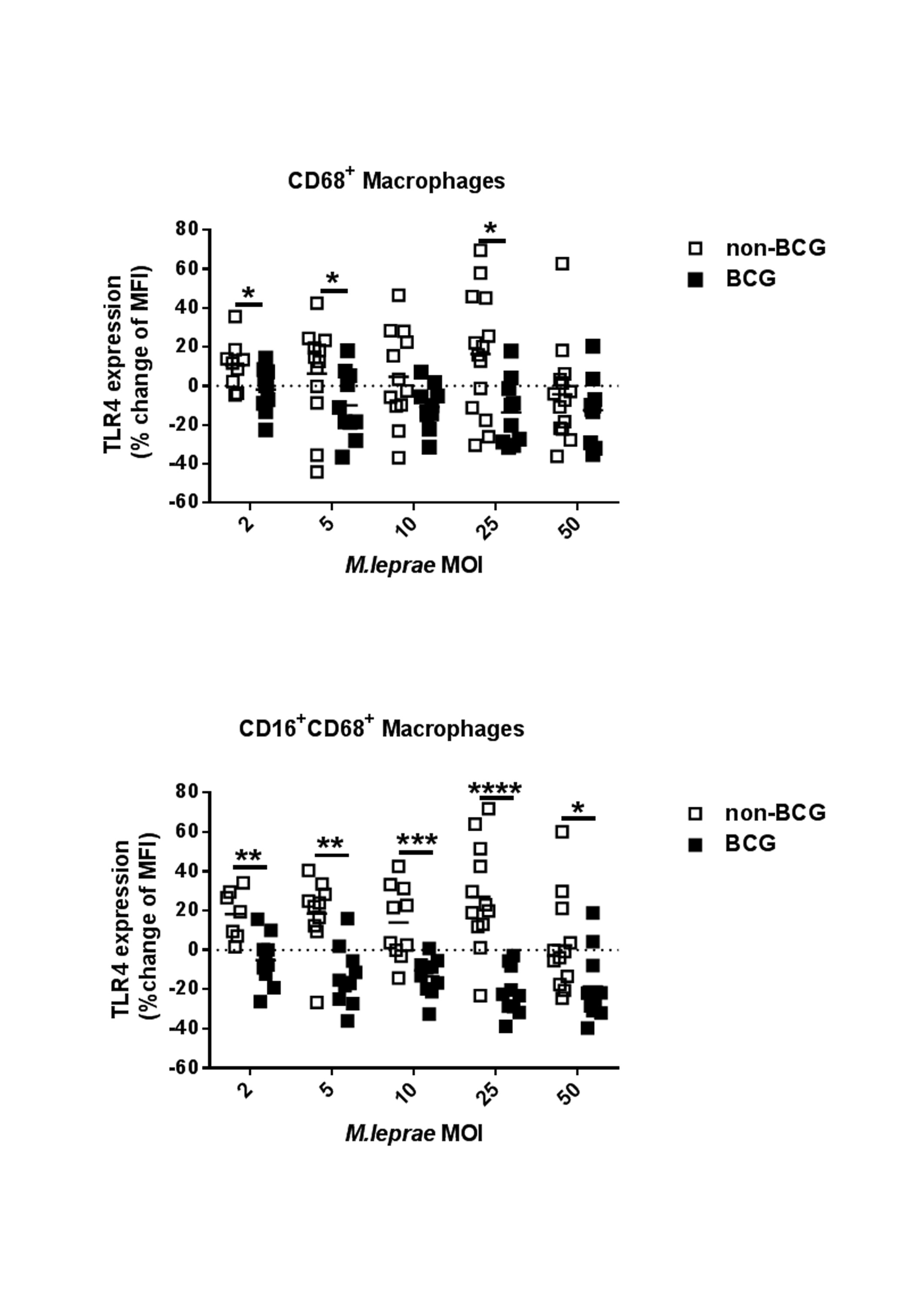

Supplement: Supplementary Image 1 — TLR4 expression in macrophages is modulated in BCG-vaccinated and non-BCG vaccinated healthy volunteers after exposure to killed M. leprae. Percentage change of Median Fluorescence Intensity (MFI) indicating expression of TLR4 compared to unstimulated control (medium) after incubation of macrophages with increasing concentrations of killed M. leprae for 18 h. Using multicolor flow cytometry, macrophages were gated as CD68+ cell population. The upper scatter plot shows the total % change in TLR4 expression compared to controls in macrophages from non-BCG vaccinated (white squares) and BCG-vaccinated (black squares) healthy volunteers, after incubation with increasing MOI of killed M. leprae. The lower scatter plot shows the subgroup analysis of percentage change of TLR4 MFI for the CD16+CD68+ macrophage population. Graphs summarize (n = 8) experiments with a total of 14 non-BCG vaccinated healthy volunteers and 10 BCG-vaccinated healthy volunteers. Mann-Whitney tests were used to compare the non-BCG vaccinated with the BCG-vaccinated donors. *0.01 < p < 0.05; **0.001 < p < 0.01; ***0.0001 < p < 0.001; ****p < 0.0001. [file Image1.JPEG]
